# Artificial intelligence in surgical decision-making across the perioperative continuum: a scoping review

**DOI:** 10.3389/fdgth.2026.1871727

**Published:** 2026-09-03

**Authors:** Wasim I. Alghoul, Bassam Awad, Rasha A. Salama, Radwan Aloti, Ayman Agha, Wed B. Al-Shammari, Mohammed M. Ali, Khaled W. Elayyan, Hussain Azimullah, Hesham A. Hamdy, Mohamed Anas Patni, Nihal Wadid

**Affiliations:** 1 College of Medicine, RAK Medical and Health Sciences University, Ras al-Khaimah, United Arab Emirates; 2 Ajman University, Ajman, United Arab Emirates

**Keywords:** artificial intelligence, clinical decision support, machine learning, perioperative care, surgical decision-making

## Abstract

**Background:**

The role of artificial intelligence (AI) in supporting clinical decision-making across the perioperative continuum remains incompletely defined. Although the presence of many AI models that perform well in terms of their predictive performance has been established, their role in the actual surgical decision-making process in the real-world in terms of specialties and perioperative phases has not been fully mapped.

**Objective:**

To map the existing literature on the application of AI in surgical decision-making across the perioperative continuum, describe its use in preoperative, intraoperative, and postoperative phases, and identify key barriers and gaps affecting clinical implementation.

**Methods:**

A scoping review was conducted in accordance with the PRISMA-ScR guidelines. PubMed, Scopus, and Google Scholar were searched for studies evaluating AI applications in surgical decision-making. Eligible studies included primary research and evidence syntheses applying machine learning, deep learning, radiomics, or computer vision to diagnosis, risk stratification, surgical planning, intraoperative guidance, or postoperative outcome prediction. Study characteristics, perioperative phase, clinical application, AI methodology, and reported implementation barriers were extracted and charted using a standardized data form.

**Results:**

Fifty- five sources of evidence were included. Sources addressing multiple or cross-phase perioperative applications constituted the largest category, while among phase-specific applications, preoperative applications were the most frequent and primarily focused on diagnosis, risk stratification, and surgical planning. Intraoperative applications were less common and were limited by data availability, workflow integration, and real-time implementation challenges. Postoperative applications mainly addressed complication prediction, survival estimation, and recovery monitoring.

**Conclusion:**

AI in surgical decision-making is expanding rapidly, with preoperative applications showing comparatively greater evidence maturity than intraoperative and postoperative applications. However, prospective validation and real-world implementation remain limited across the perioperative continuum.

## Graphical Abstract

**Figure:**
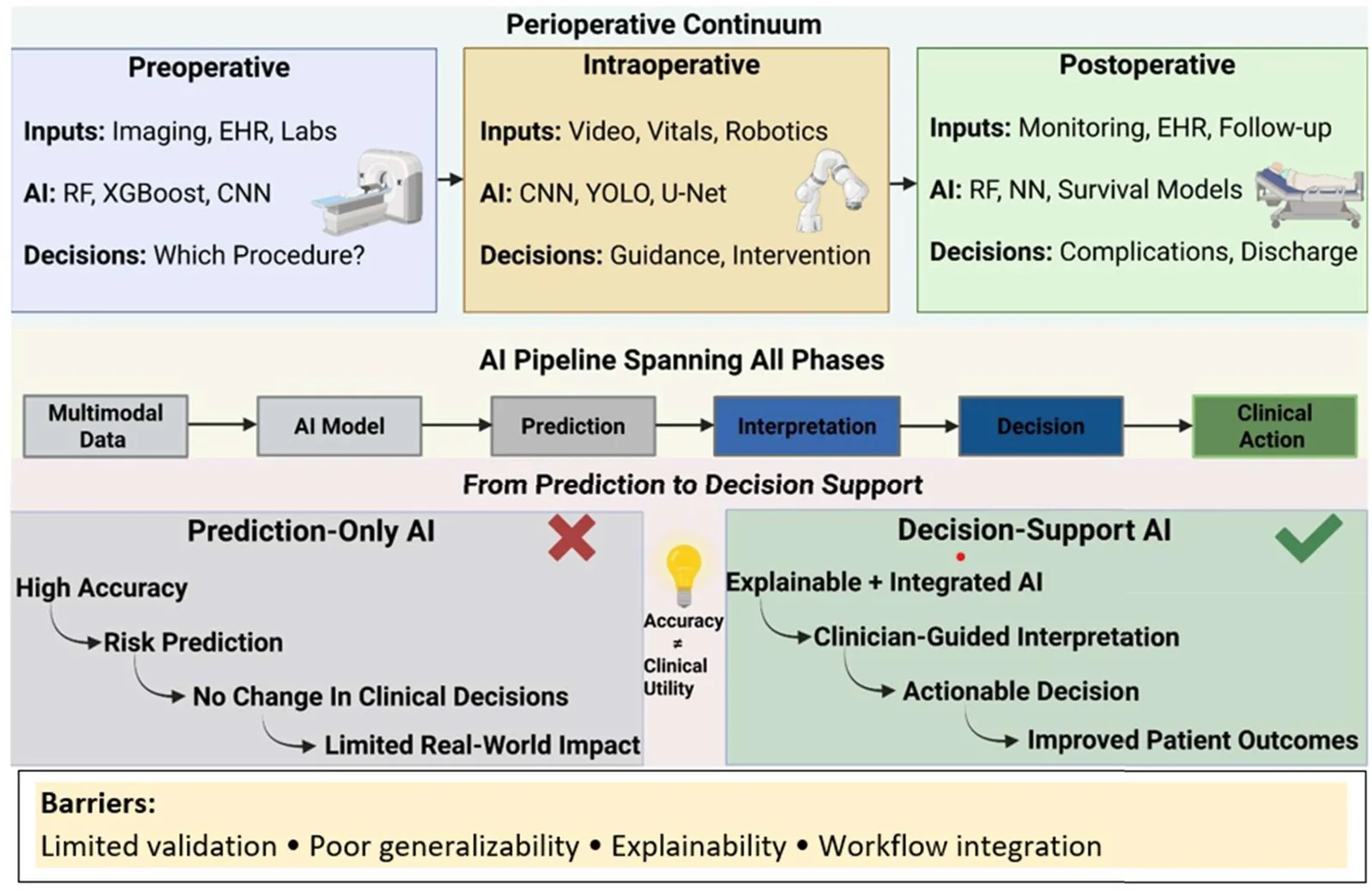
AI in surgical decision-making across the perioperative continuum.

## 1 Background

The surgical decision-making process is a high-stakes but complex process that spans the entire continuum of perioperative care, including patient selection, procedural planning, intraoperative management, and postoperative care. These decisions have traditionally been based on clinician experience, heuristic judgment, and traditional risk scores based on population-level data. Although these approaches remain the cornerstone of surgical decision-making, they are increasingly challenged by growing patient complexity, expanding treatment options, and the demand for more individualized and data-driven care. Artificial intelligence (AI) has emerged as a potentially transformative technology capable of augmenting surgical decision-making through the analysis of high-dimensional clinical data (1, 2).

Within the past decade, AI and machine-learning (ML) techniques have demonstrated impressive outcomes in predicting surgical outcomes (including complications, mortality, readmissions, and length of stay) (1, 3). However, predictive accuracy is not enough to guarantee clinical usefulness. The decisions made in surgery must be understandable, responsible, and must be integrated into the workflow of practitioners, particularly when the decisions made are irreversible or high-risk decisions. Poorly calibrated or opaque AI models may inadvertently promote inappropriate surgical avoidance, overtreatment, or delayed intervention (4). Therefore, an important distinction exists between AI systems developed primarily for prediction and those intended to influence or support clinical decision-making, although both may contribute to perioperative care. Each phase of the perioperative continuum presents distinct decision-making challenges. Preoperative decision-making involves determining surgical candidacy, selecting the most appropriate treatment strategy, and planning the operative approach. Similarly, intraoperative decisions are time-sensitive and often must be made under conditions of uncertainty while rapidly interpreting physiological changes and procedural indicators. Postoperative clinicians have to decide on disposition, monitoring, and early intervention in response to the changing status of patients. The parameters of AI systems that may be considered vary depending on the stage, with latency, interpretability, robustness, and error-tolerance being the most important parameters to be considered (2, 4). Earlier AI in surgery had been largely risk stratification focused, and the models developed had been based upon predicting adverse events as a function of preoperative variables (3, 5). These tools, in spite of having small improvements as compared to the conventional regression-based scores, were vague in their effect on the final decision-making process. Surveys of surgical ML models report that most investigators have concluded by reporting measures of discrimination without demonstrating the impact of predictions on clinician behavior or patient outcomes (1, 6). This inability to connect has resulted in the cynicism of surgeons regarding the practical usefulness of AI despite the sound methodological developments. Recent literature increasingly emphasizes the role of AI as a decision-support tool rather than an autonomous decision-maker. Decision-support systems are designed to supplement and contextualize clinician judgment while maintaining human oversight. The ethical and regulatory requirements of surgical practice mean that responsibility for clinical decisions cannot be delegated to an algorithm, which would allow incorporating AI into shared decision-making, where patients can take a position in the discussions about the risk and benefit of different treatment options (2, 4). Another key challenge highlighted in the early AI–surgery literature is generalizability. Most ML models are only trained using data at a single center and may not be effective when applied to new institutions with different patient populations, surgery, or perioperative processes (6, 7). This has been identified as a particular concern in the sphere of surgery, where local resources and expertise can be central to its decision-making. Without adequate external validation and recalibration, AI-assisted decisions may be unreliable when applied in different clinical settings (4, 7).

Interpretability and transparency are also concerning in surgical decision-making. Surgeons must be able to explain and justify AI-supported recommendations to patients and multidisciplinary teams. Black-box models may be unsuitable for high-stakes surgical decisions because the rationale underlying their predictions is often difficult to interpret. Several methodological reviews emphasize that interpretable models and clinically meaningful explanation methods are essential prerequisites for trust and adoption in surgical practice (1, 8).

Regulatory and governance considerations also influence the adoption of AI in surgical practice. Although regulatory approval has begun to emerge for selected applications, most AI systems reported in the surgical literature remain at the preclinical or developmental stage (9). The need to distinguish between such tools that merely inform the clinicians and those that actively influence the choices that are made by clinicians is an issue that was highlighted in the initial regulatory deliberations (4, 9).

Prospective evaluation is another recurring theme in the AI–surgery literature. Although retrospective validation is an important first step, it alone is insufficient to establish clinical utility. Ultimately, AI-supported decisions must demonstrate improvements in patient outcomes, reduction of complications, workflow efficiency, or other meaningful clinical endpoints. Consequently, prospective impact studies and real-world evaluations remain essential for clinical translation (6, 10).

Lastly, the ethical aspects such as bias, equity, and fairness are also becoming known as part of AI-supported decision-making. Patient equity is greatly affected by the ramifications of access to care, the time of intervention, and the resources to utilize during the postoperative period. When AI models are trained on lopsided or incomplete information, they may be able to support or further increase differences. Background reviews emphasize the importance of performing an audit of AI systems on demographic and clinical subgroups and applying ethical control to the process of model development and implementation (8, 11).

In summary, the existing evidence highlights both the potential and the limitations of artificial intelligence (AI) in supporting surgical decision-making. Although technical capabilities have advanced rapidly, translation into clinically reliable and implementable decision-support tools remains uneven across perioperative settings. Much of the literature has focused on predictive performance, while less attention has been given to how AI influences real-world clinical decisions and implementation. Therefore, a scoping review is warranted to comprehensively map the current evidence on AI-supported surgical decision-making across the perioperative continuum, identify areas of maturity, and highlight barriers and gaps affecting clinical translation.

To address these gaps, this scoping review was guided by four key research questions: (1) What types of AI applications are used across the perioperative phases of surgical care? (2) How does AI contribute to decision-making in preoperative, intraoperative, and postoperative settings? (3) How does the performance of AI-based approaches compare with traditional clinical or statistical methods? and (4) What are the main barriers to the clinical implementation of AI in surgical practice?

To this end, this review aimed to map the existing literature on AI in surgical decision-making across the entire perioperative continuum, describe its current uses in various phases, the most important decisions aided by AI, and gaps/barriers to clinical translation.

## 2 Methods

### 2.1 Study design and reporting guidelines

This scoping review was conducted in accordance with the Preferred Reporting Items for Systematic Reviews and Meta-Analyses extension for Scoping Reviews (PRISMA-ScR).

### 2.2 Search strategy

A comprehensive literature search was conducted in PubMed/MEDLINE, Scopus, and Google Scholar to identify studies evaluating the application of artificial intelligence (AI) to surgical decision-making across the perioperative continuum. The searches were performed on 15 February 2026 and were restricted to English-language publications published between 1 January 2019 and 31 December 2025.

The search strategy was intentionally focused on studies explicitly addressing AI-supported surgical or perioperative decision-making rather than the broader literature on artificial intelligence in medicine. Database-specific search strings combined terms for artificial intelligence or machine learning with surgical or perioperative terms and decision-support concepts, including decision-making, prediction, planning, and clinical outcomes. Search syntax and field restrictions were adapted to the requirements of each database.

The PubMed/MEDLINE search retrieved 1,114 records, the Scopus search retrieved 1,042 records, and the Google Scholar search retrieved 744 records, yielding 2,900 records before duplicate removal and relevance refinement. The complete executable search strategies for PubMed/MEDLINE, Scopus, and Google Scholar, including the full Scopus Advanced Search syntax, field restrictions, Boolean operators, publication-date limits, language restrictions, and initial retrieval counts, are provided verbatim in Supplementary Table S2.

Because the review was specifically focused on AI applications with a clinically relevant surgical or perioperative decision-support role, a predefined relevance-refinement step was applied before formal title and abstract screening. Records that did not demonstrate an eligible AI application, a relevant surgical or perioperative context, or a potential clinical decision-support role were removed at this stage. A total of 2,124 records were removed during relevance refinement. Six duplicate records were subsequently removed, leaving 770 records for formal title and abstract screening.

For Google Scholar, results were screened according to relevance ranking using the predefined eligibility criteria. Reference lists of eligible studies and relevant review articles were additionally examined to identify potentially relevant supporting sources.

### 2.3 Eligibility criteria

Studies were eligible if they: (1) evaluated an application of artificial intelligence relevant to surgical decision-making or perioperative clinical decision support; (2) addressed a preoperative, intraoperative, postoperative, or cross-phase clinical application; (3) involved machine learning, deep learning, radiomics, computer vision, natural language processing, large language models, or another relevant AI-based approach; (4) had a clinically relevant role in diagnosis, risk stratification, patient or treatment selection, operative planning, intraoperative guidance or monitoring, postoperative prediction, or another decision-support function; and (5) were published in English between 1 January 2019 and 31 December 2025.

Eligible evidence sources included primary studies, including prospective, retrospective, cohort, case-control, validation, and comparative studies, as well as systematic reviews, meta-analyses, scoping reviews, and narrative reviews relevant to the objectives of the scoping review. Studies were excluded if they lacked a clear surgical or perioperative clinical application, did not involve an eligible AI approach, lacked a clinically relevant decision-support role, provided insufficient methodological detail, represented duplicate publications, or were conference abstracts, editorials, or letters without sufficient methodological information.

Both primary and secondary evidence sources were retained because the objective of this scoping review was to map the breadth, maturity, and clinical implementation of AI-supported surgical decision-making rather than to estimate a pooled intervention effect. Evidence type was recorded during data charting and considered explicitly during narrative synthesis to reduce the risk of inappropriate overlap between primary studies and evidence syntheses. For articles published online ahead of print, the electronic publication date was used to determine eligibility.

For the purposes of this review, clinical decision support was defined as an AI-derived output that could inform a specific clinician choice or action within the perioperative pathway. Eligible decision points included surgical candidacy or patient selection, diagnostic or staging decisions that directly influence surgical management, treatment or procedure selection, operative planning, intraoperative identification, guidance or intervention, and postoperative monitoring, escalation, disposition, or follow-up planning. Prediction-only applications were considered eligible only when the predicted outcome had a clear and clinically plausible link to one of these perioperative decisions. Studies limited to technical performance, education or simulation, administrative workflow, or general discussion of AI without an identifiable clinical decision were not considered direct decision-support evidence.

### 2.4 Study selection

**Figure 1:**
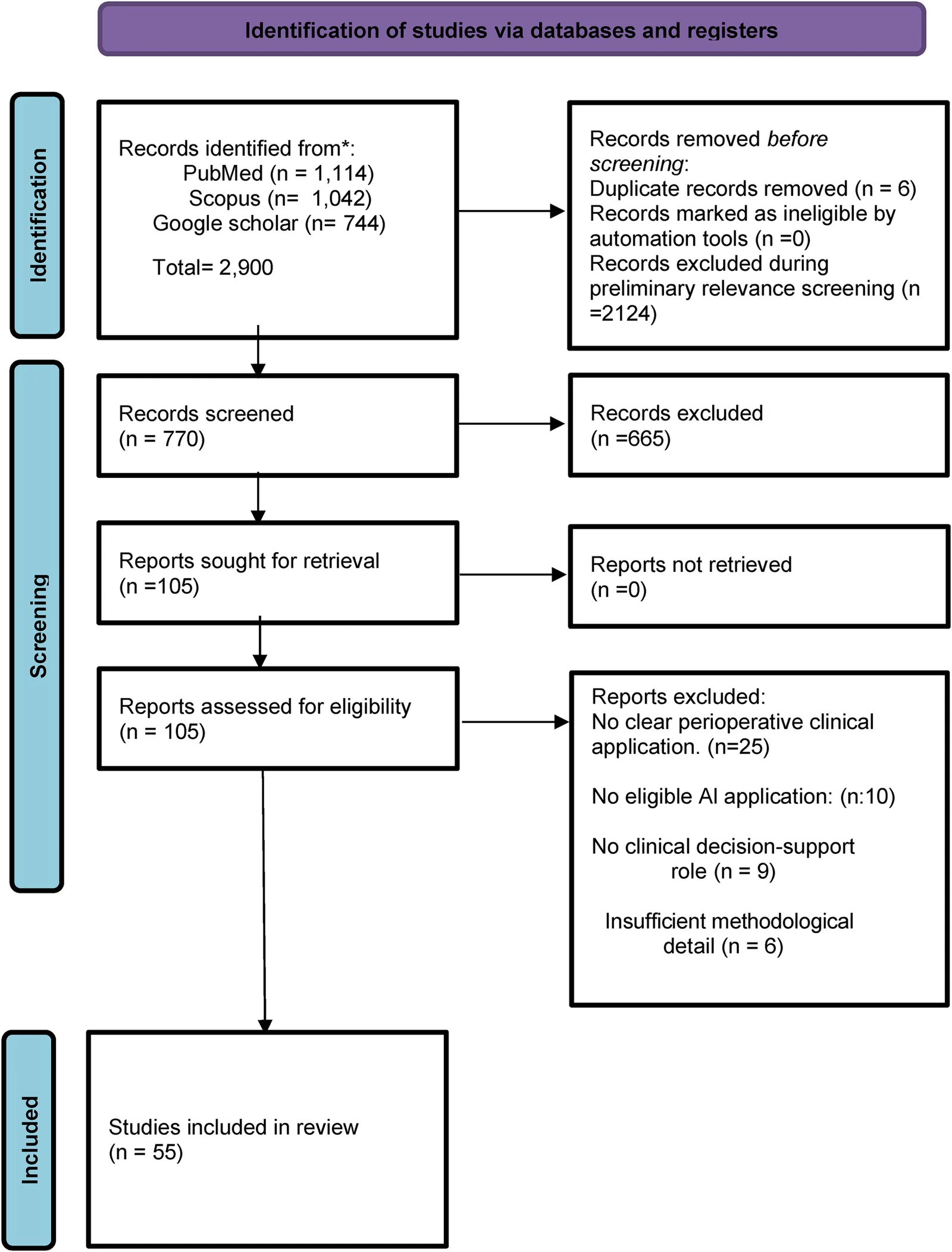
.

The study-selection process was conducted in sequential stages and is summarized in the PRISMA-ScR flow diagram (Figure 1). The database searches initially identified 2,900 records: 1,114 from PubMed/MEDLINE, 1,042 from Scopus, and 744 from Google Scholar.

Before formal title and abstract screening, 2,124 records were removed during the predefined relevance-refinement stage because they did not sufficiently correspond to the focused review question. Six duplicate records were also removed, leaving 770 records for formal title and abstract screening.

Two reviewers independently screened the titles and abstracts of these 770 records against the predefined eligibility criteria. A total of 665 records were excluded at this stage, and 105 reports were sought for full-text retrieval. All 105 reports were successfully retrieved and independently assessed for eligibility by the same reviewers.

Following full-text assessment, 50 reports were excluded: 25 because they lacked a clear perioperative clinical application, 10 because they did not involve an eligible AI application, nine because they lacked a clinical decision-support role, and six because they provided insufficient methodological detail. Consequently, 55 sources of evidence were included in the final qualitative synthesis.

Disagreements between the two reviewers at any stage were resolved through discussion and consensus. Where consensus could not be reached, a third reviewer was consulted for adjudication.

### 2.5 Data charting and extraction

Data were charted independently by two reviewers using a standardized data-charting form developed specifically for this review. Before full extraction, the form was piloted on a sample of studies and refined to ensure consistency.

The following information was extracted from each study: author, year of publication, country, study design, surgical specialty, perioperative phase, clinical application, AI methodology, data source, validation approach, reported performance metrics, decision-making role, implementation characteristics, and reported barriers to clinical translation.

Extracted data were cross-checked for accuracy and completeness. Any discrepancies were resolved through discussion and consensus between reviewers, with adjudication by a third reviewer when required.

In addition to clinical and methodological characteristics, each included source was classified as either primary research or secondary evidence. Primary studies were used to characterize individual AI applications, study-level outcomes, validation approaches, and evidence of clinical implementation. Secondary evidence sources, including systematic, scoping, and narrative reviews, were used to contextualize broader patterns across specialties, identify recurring implementation barriers, and map research gaps. Primary and secondary evidence were therefore synthesized separately and were not treated as equivalent independent observations. Where secondary reviews summarized primary studies that overlapped with other included evidence, they were used for contextual synthesis only and were not interpreted as additional independent confirmation of the same findings. No quantitative pooling across primary and secondary evidence was performed.

Translational readiness was assessed descriptively during evidence synthesis based on four implementation-related domains: (1) validation maturity, including external and prospective validation; (2) workflow integration feasibility; (3) clinician acceptability and trust; and (4) evidence of real-world implementation or deployment. Rather than applying a formal numerical score or rigid categorical threshold, readiness was summarized qualitatively according to the overall maturity of the evidence for each source. Descriptors such as early, moderate, or more advanced readiness were used to reflect the degree of validation, clinical integration, and implementation demonstrated or discussed in the source. For secondary evidence sources, readiness assessments reflected the overall maturity of the evidence summarized within the review rather than a single AI model. The study-level basis for these assessments is provided in Supplementary Table S1.

Implementation barriers were identified and charted using a pragmatic sociotechnical framework. Reported barriers were categorized into data availability and quality, validation maturity, workflow integration, explainability, clinician trust, regulatory readiness, and real-world implementation challenges.

The complete dataset of charted study characteristics is provided in Supplementary Table S1.

### 2.6 Protocol and registration

No formal protocol was registered for this scoping review. The review was conducted in accordance with the PRISMA-ScR reporting guideline, and the methodology was predefined by the review team before study selection and data charting.

## 3 Results

### 3.1 Study selection

The study-selection process is summarized in the revised PRISMA-ScR flow diagram (Figure 1). The database searches initially identified 2,900 records, comprising 1,114 records from PubMed/MEDLINE, 1,042 from Scopus, and 744 from Google Scholar. Before formal screening, 2,124 records were removed during the predefined relevance-refinement step and six duplicate records were removed, leaving 770 records for title and abstract screening.

Of the 770 records screened, 665 were excluded, and 105 reports were sought and successfully retrieved for full-text assessment. Following full-text review, 50 reports were excluded: 25 because they lacked a clear perioperative clinical application, 10 because they did not involve an eligible AI application, nine because they lacked a clinical decision-support role, and six because of insufficient methodological detail. Consequently, 55 sources of evidence were included in the final qualitative synthesis.

### 3.2 Characteristics of included studies

The 55 included sources of evidence comprised 8 primary research studies and 47 secondary evidence sources. The eight primary studies were Bang et al. (12), Ma et al. (13), Zeng et al. (14), Yu et al. (15), Yang et al. (16), Ding et al. (17), Gong et al. (18), and Merali et al. (19). These included retrospective and prospective modeling studies, multicenter comparative studies, diagnostic-model studies, and model-development and validation studies. The remaining 47 sources comprised systematic reviews, scoping reviews, narrative reviews, meta-analyses, and other evidence syntheses.

Zeng et al. (14) was published online on 10 September 2025 and subsequently assigned to the journal's 2026 issue. Consistent with the predefined eligibility criteria, the electronic publication date was used to determine eligibility.

The two evidence types were interpreted separately. Study-level conclusions regarding model validation, prospective evaluation, and clinical deployment were derived primarily from the eight primary studies, whereas secondary evidence was used to characterize broader trends, specialty-level patterns, implementation barriers, and areas of research uncertainty. The secondary evidence sources were not treated as 47 independent confirmations of the findings reported by the primary studies.

The studies encompassed a broad range of surgical specialties, including orthopedics, neurosurgery, gastrointestinal surgery, oncology, maxillofacial surgery, and anesthesiology. The included sources were published within the predefined eligibility period from 1 January 2019 to 31 December 2025, reflecting the growing integration of artificial intelligence (AI) into surgical practice.

Across the included studies, AI applications were primarily used for diagnostic support, risk stratification, surgical planning, intraoperative guidance, and postoperative outcome prediction. Machine learning and deep learning were the most frequently employed methodologies. Detailed characteristics of all included studies are provided in Supplementary Table S1, while representative examples illustrating the diversity of AI applications, validation approaches, and implementation challenges are presented in Table 1.

**Table 1:** Representative examples of AI applications in surgical decision-making across the perioperative continuum.

Study	Evidence type	Specialty	Phase	Decision supported	AI method	Key outcome	Validation	Key barrier
Kokkotis et al. (22)	Systematic Review	Orthopedics	Postoperative	Functional recovery assessment	ML	Improved outcome prediction	Not specified	Small datasets, heterogeneity
Bianco et al. (23)	Systematic Review	Pediatric Surgery	Preoperative & Postoperative	Diagnosis & risk stratification	ML	Improved prediction tools	Limited external validation	Implementation challenges
Zhou et al. (24)	Systematic Review	GI/Pancreatic Surgery	Preoperative	Severity prediction	ML	Accurate complication prediction	Limited	Retrospective bias
Chevalier et al. (32)	Systematic Review	Multispecialty	All phases	General decision support	AI/ML	Decision-making potential	Not specified	Ethical & regulatory issues
Buchlak et al. (35)	Systematic Review	Neurosurgery	Pre/Intra	Diagnosis & planning	ML	Enhanced decision support	Limited	Data heterogeneity
Yu et al. (15)	Primary Study (Multicenter)	Breast Oncology	Preoperative	Lymph node prediction	Radiomics/ML	Improved surgical planning	External	Generalizability
Gong et al. (18)	Primary Validation Study	Gastroenterology/Surgery	Intraoperative/diagnostic	Clinical decision support	AI/DL	Real-time decision support	External validation	Workflow integration
Zeng et al. (14)	Primary Comparative Study	Colorectal Surgery	Preoperative	Guideline adherence	LLM	Comparable to clinicians	Comparative validation	Clinical implementation
Merali et al. (19)	Primary Cohort Study	Neurosurgery	Postoperative	Outcome prediction	ML	Improved prognostication	Internal validation	External validity

### 3.3 Distribution of AI applications across perioperative phases

**Figure 2:**
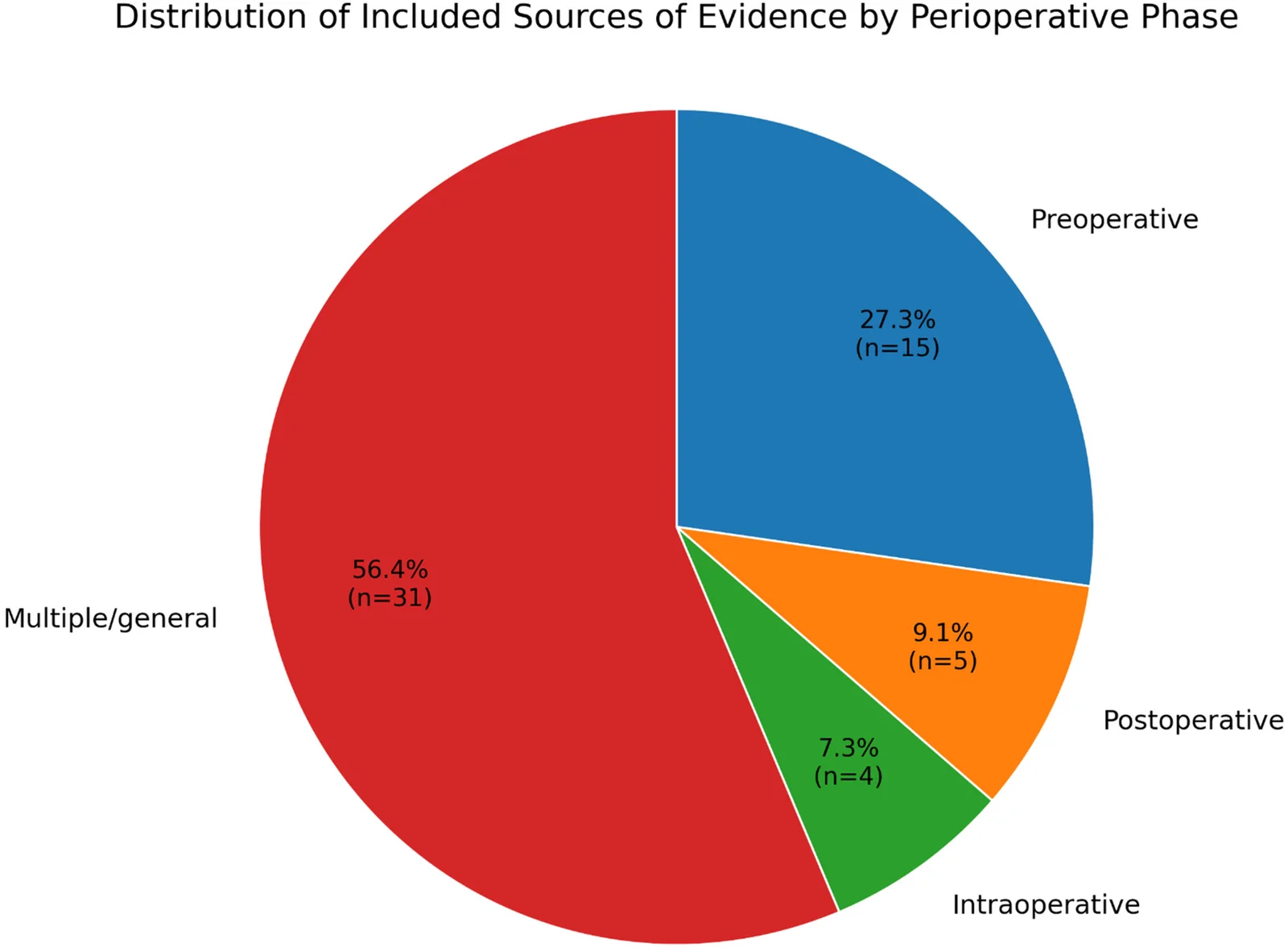
Distribution of included sources of evidence by perioperative phase of artificial intelligence application. Percentages are based on *n* = 55 and may not total 100% because of rounding. These values describe the distribution of included publications and do not represent 55 independent primary datasets.

The included sources of evidence were categorized according to the predominant perioperative phase of AI application (Figure 2). Of the 55 included sources, 15 (27.3%) were classified as primarily preoperative, focusing on diagnosis, risk stratification, patient selection, and surgical planning.

Five sources (9.1%) were classified as primarily postoperative, addressing complication prediction, functional outcomes, recurrence, mortality, and recovery monitoring. Four sources (7.3%) were classified as primarily intraoperative, focusing on real-time decision support, anatomical recognition, endoscopic or operative guidance, and operating-room applications.

The largest category comprised 31 sources (56.4%) that addressed multiple perioperative phases or provided general or cross-phase evidence relating to AI-supported surgical care. This distribution reflects the broad, cross-continuum nature of much of the available literature rather than concentration within a single perioperative phase.

These counts represent the distribution of included publications rather than independent underlying datasets; secondary evidence sources may summarize overlapping primary studies and should not be interpreted as independent replications.

A summary of key outcomes, barriers, and translational readiness across perioperative phases is presented in Table 2.

**Table 2:** Implementation outcomes and translational readiness across perioperative phases.

Phase	Clinical applications	Implementation strengths	Key adoption barriers	Translational readiness
Preoperative	Diagnosis, risk stratification, surgical planning	Strong predictive performance, growing external validation, easier integration into existing workflows	Limited prospective evaluation, explainability concerns, generalizability issues	Moderate
Intraoperative	Real-time guidance, anatomical recognition, physiologic monitoring	Potential for immediate decision support and enhanced procedural safety	Workflow disruption, latency requirements, clinician trust, limited prospective validation	Low
Postoperative	Complication prediction, survival prediction, recovery monitoring	Availability of longitudinal outcome data and established prediction models	Limited integration into follow-up pathways, heterogeneity of outcomes, insufficient prospective studies	Low–Moderate

Phase-level translational readiness represents a qualitative and relative synthesis of the available evidence, considering validation maturity, workflow integration feasibility, clinician acceptability, and evidence of real-world implementation. These descriptors indicate comparative implementation maturity within the included literature and should not be interpreted as evidence of established clinical readiness or routine deployability.

### 3.4 Implementation outcomes and translational readiness

Analysis of implementation-related findings across the included studies identified several recurring domains influencing the adoption of AI in surgical decision-making. Feasibility was greatest in preoperative settings, where AI tools could be more readily integrated into existing diagnostic and planning workflows, whereas real-time intraoperative environments presented substantial operational challenges. Workflow integration emerged as a major barrier for intraoperative and postoperative applications, particularly where AI outputs required seamless incorporation into clinical pathways and electronic health record systems.

Explainability and clinician trust were recurrent concerns across all perioperative phases, reflecting the need for transparent and interpretable AI-supported recommendations. Validation maturity also remained limited, with many studies relying on retrospective datasets and reporting insufficient external or prospective validation. Regulatory readiness was infrequently addressed, and only a small proportion of studies discussed regulatory approval pathways or implementation frameworks. Acceptability was generally more favorable when AI was positioned as a decision-support tool that augmented clinician judgment rather than replacing it.

### 3.5 Evidence maturity and readiness for clinical deployment

The included studies revealed substantial variation in the maturity of AI applications across perioperative phases. Preoperative applications, particularly those related to diagnostic support, risk stratification, and surgical planning, appeared relatively more mature than intraoperative and postoperative applications. This relative maturity reflected larger datasets and more frequent validation efforts in some preoperative applications; however, prospective evaluation and evidence of real-world clinical implementation remained limited. These applications were generally supported by larger datasets, more frequent validation efforts, and clearer integration into existing clinical workflows.

A distinction was observed between studies evaluating predictive performance and those assessing the use of AI within clinical decision-making processes. Most studies focused on predicting outcomes, complications, or risk profiles. Comparatively fewer studies examined how AI outputs were incorporated into treatment selection, surgical planning, intraoperative guidance, or postoperative management decisions. As a result, much of the existing literature emphasizes model performance rather than the real-world impact of AI on clinical decision-making.

At the level of primary evidence, validation maturity varied considerably across the eight studies. Although several studies reported favorable predictive performance, prospective evaluation remained uncommon, and most evidence was derived from retrospective analyses. Validation requirements differed according to the intended application. Some studies reported external validation, particularly for models intended for broader deployment, whereas locally developed tools often focused on institution-specific validation and implementation contexts. Overall, relatively few studies assessed AI tools in prospective clinical environments or evaluated their impact on real-world decision-making. Among the eight primary studies, two reported external validation. Three studies (Yu et al., Yang et al., and Merali et al.) employed prospective study designs, while **Gong et al. employed prospective evaluation through clinical validation of the AI-supported decision-making tool**. Consequently, four primary studies contributed prospective evidence overall. Most remaining primary studies relied on retrospective datasets and internal validation approaches. Evidence of prospective real-world implementation remained limited. Findings from the 47 secondary evidence sources were used to contextualize these study-level observations but were not counted as additional independent validation evidence.

From a translational perspective, preoperative applications demonstrated relatively greater implementation maturity than intraoperative and postoperative applications, particularly because some had stronger validation profiles and fewer real-time workflow requirements. However, this should not be interpreted as evidence of readiness for routine clinical deployment, as prospective evaluation, external validation, and real-world implementation remained limited. In contrast, intraoperative applications remained at an earlier stage of implementation maturity because of challenges related to workflow integration, clinician trust, and the need for reliable real-time performance. Importantly, intraoperative AI studies were also limited by restricted access to high-quality annotated surgical video datasets. Data governance restrictions, annotation burden, and the lack of standardized repositories emerged as major barriers to the development and validation of computer vision models, often representing a greater challenge than computational performance itself.

Postoperative applications showed emerging but still limited implementation maturity, with promising predictive performance but insufficient prospective validation and uncertainty regarding long-term clinical impact. Recent studies have increasingly explored the use of large language models for documentation, discharge summaries, patient communication, and care-transition support. However, despite promising early developments, real-world implementation remains limited, highlighting a persistent gap between technological innovation and routine clinical adoption.

The study findings indicate that the field remains dominated by predictive AI models, whereas clinically validated decision-support systems capable of influencing routine surgical decision-making remain comparatively limited. While several AI systems have demonstrated performance comparable to or exceeding that of clinicians in narrowly defined tasks, evidence supporting widespread autonomous deployment in surgical decision-making remains limited.

## 4 Discussion

This scoping review synthesizes 55 sources of evidence examining the role of artificial intelligence (AI) in surgical decision-making across the perioperative continuum, building on recent comprehensive reviews that highlight its expanding use in predicting surgical outcomes and informing clinical decisions (20). The review addresses four key research questions related to application types, decision-making roles, comparative performance, and implementation barriers.

**Interpretation of clinical maturity and translational readiness was weighted toward the eight primary studies, while the 47 secondary evidence sources were used to contextualize broader patterns and were not regarded as equivalent independent evidence.**

### 4.1 Preoperative phase

The risk stratification, diagnosis, patient selection, and surgical planning of AI applications (RQ1) were most commonly used during the preoperative stage across various specialties (15–18, 21–31). These applications represent comparatively more mature and diverse uses of AI within the included literature, particularly in imaging-based oncology, neurosurgical planning, and orthopedic outcome prediction; however, prospective and real-world evidence remains limited. Regarding decision-making (RQ2), AI can play a role in critical clinical decision-making, including determining whether an individual is surgically candid or ineligible, the stage of disease, treatment strategies, and optimizing procedural planning. These applications facilitate more personalized and data-driven clinical decision-making by integrating clinical, imaging, and laboratory information (32–36).

On the issue of performance (RQ3), most of the studies reported greater predictive accuracy with many variables compared to more traditional statistical models in the high-dimensional data analysis (radiomics and imaging analysis) issue. Nevertheless, the gains would not be evidenced in all areas, and in those areas where gains were observed, they were limited to the ones related to colorectal and orthopedics (37, 38).

Despite promising results, several barriers (RQ4) persist. These include reliance on retrospective datasets, limited external validation, heterogeneity of study populations, and lack of generalizability across institutions. Ethical concerns, including bias and explainability, further limit clinical adoption (12, 33, 34).

### 4.2 Intraoperative phase

Real-time interpretations of images, anatomical recognition, surgical workflow analysis, and physiologic monitoring were other less popular and common uses of AI in the intraoperative period (21, 32, 39). The scope of intraoperative AI applications remains more limited than that of preoperative applications and continues to face substantial technical challenges.

AI has the potential to support real-time intraoperative decision-making through tissue identification, surgical navigation, and physiological risk assessment. They are particularly applicable in procedures that are minimally invasive and imaging-based.

Regarding performance (RQ3), several studies reported improved anatomical recognition and a reduced incidence of intraoperative complications, particularly in laparoscopic surgery and anesthesiology (40, 41). However, there is still much more evidence to be concerned with the retrospective/proof-of-concept experiments and few prospective or real-world validations.

At this stage, implementation barriers remain substantial. Key implementation barriers include real-time data processing requirements, latency constraints, workflow integration challenges, and limited clinician trust. The requirement for rapid and interpretable decision support in high-stakes surgical environments further complicates implementation (40–42).

A major implementation barrier in intraoperative AI is access to suitable data. Computer vision models require large volumes of high-quality, annotated surgical videos; however, such datasets are often difficult to obtain because of privacy concerns, governance restrictions, annotation burden, and lack of standardized repositories. Therefore, data access and availability represent primary barriers, while real-time processing and latency are secondary technical challenges.

### 4.3 Postoperative phase

Most of the applications of AI implemented after the operation (RQ1) had a predominant and major focus on complications prediction, survival prediction, recurrence prediction and recovery prediction (13, 19, 21, 32, 33, 43–50). This phase benefits from the availability of rich longitudinal outcome data, which can be used to develop predictive models across diverse clinical settings.

Regarding decision-making (RQ2), AI can support decisions related to postoperative monitoring, follow-up planning, complication risk stratification, and individualized recovery pathways. These tools may facilitate more proactive and personalized postoperative care.

In terms of the performance (RQ3) factor, AI models have proved to have acceptable to good predictive accuracy. However, as demonstrated by colorectal and other outcome prediction systems, AI has not necessarily worked better than the standard statistical model, which can also indicate that its advantage is the fact that it could combine and make the most of the complex variables (37, 38).

It is not essentially different from other stages; the barriers (RQ4) are not any different, most importantly in the presence of prospective studies. Others are the entry into the clinical follow-up routes, and the potential bias to influence the decision of postoperative care (34, 51).

Recent advances in large language models have expanded interest in postoperative applications, particularly clinical documentation, discharge summaries, patient communication, and care-transition support. However, adoption in routine postoperative workflows remains slow, reflecting a development–implementation gap between technical capability and clinical deployment.

### 4.4 Variation across surgical specialties

Applications of artificial intelligence and maturity by surgical specialty differed significantly in the studies included. Fields with the greatest number of applications were in imaging-intensive areas like oncology, gastrointestinal surgery, and neurosurgery, with the most advanced applications seen in the areas of diagnosis, staging, and treatment planning (26–29, 52–56). Other specialties, such as orthopedics and general surgery, were more heterogeneous but broader in their use and mostly in the aspects of prediction of outcomes and resource optimization (36, 57, 58).

Craniofacial, maxillofacial, and aesthetic surgery provided particular applications in the fields associated with simulation, anatomy modeling, and anticipation of the outcomes, and AI contributed to them (59–65). Minimal developments in intraoperative applications had been validated in minimally invasive and image-guided surgery, such as laparoscopic surgery and endoscopic surgery, but not in other specialties (39, 40).

Overall, these findings suggest that AI-based constructions and the emergence of robots heavily rely on the presence of accessible data, the complexity of the solutions, and the degree to which surgery decision-making is concerned with imaging and measurable solutions.

### 4.5 Overall interpretation

The present synthesis suggests a relative gradient of evidence maturity across the perioperative continuum. Preoperative applications appear comparatively more mature than postoperative and intraoperative applications; however, evidence supporting routine clinical implementation remains limited across all three phases. Importantly, the literature remains dominated by predictive models rather than fully implemented decision-support systems capable of demonstrably influencing clinical decisions and patient outcomes.

Although many studies position AI as a decision-support tool, the role of AI in surgical decision-making should be interpreted more broadly. Some AI systems may outperform clinicians in narrowly defined prediction or classification tasks. However, surgical decision-making often involves patient preferences, ethical considerations, contextual judgment, accountability, and uncertainty that extend beyond algorithmic performance. Therefore, the current evidence supports a spectrum of human–AI collaboration rather than a universal model of either full automation or clinician-only decision-making.

In addition to validation, workflow integration, and explainability, affordability and accessibility are important determinants of successful implementation. AI applications have also been explored for operating room workflow optimization, resource allocation, and perioperative management, highlighting the importance of implementation considerations beyond predictive performance alone (66). AI applications in total and unicompartmental knee arthroplasty have also been reviewed (67). Consequently, implementation decisions should consider not only predictive accuracy but also cost-effectiveness, scalability, accessibility, infrastructure requirements, and feasibility within routine clinical practice. These considerations may be particularly important in resource-constrained healthcare settings, where limited access to advanced computational infrastructure and technical expertise may restrict the adoption of otherwise promising AI solutions.

Overall, preoperative applications appear comparatively closer to clinical translation than intraoperative and postoperative applications because of relatively stronger validation profiles and fewer real-time workflow requirements. Nevertheless, the available evidence remains insufficient to characterize these applications as broadly ready for routine clinical deployment, particularly given the limited prospective and real-world evaluation. In contrast, intraoperative applications remain constrained by challenges related to workflow integration, real-time processing requirements, data availability, and clinician trust, thereby limiting their readiness for widespread clinical deployment. Postoperative applications occupy an intermediate position, with promising predictive capabilities but limited prospective validation and real-world implementation. Together, these findings suggest that future efforts should focus not only on improving model performance but also on addressing practical implementation considerations that facilitate sustainable and equitable adoption in clinical practice.

## 5 Conclusion

This scoping review mapped the current evidence on artificial intelligence applications in surgical decision-making across the perioperative continuum. Preoperative applications demonstrated comparatively greater evidence maturity than intraoperative and postoperative applications; however, the evidence supporting real-world implementation remained limited across all perioperative phases.

Although AI has demonstrated strong predictive performance in many surgical domains, evidence supporting the routine deployment of AI-enabled decision-support systems remains limited. Future progress will depend not only on improving model performance but also on strengthening validation, demonstrating clinical utility, integrating AI into clinical workflows, and evaluating real-world impacts on patient outcomes and decision-making.

AI has considerable potential to enhance surgical decision-making across the perioperative continuum. Although applications span multiple phases of care, much of the evidence remains at an early or intermediate stage of translation, with limited prospective validation and real-world implementation. Future research should prioritize robust multicenter evaluation, regulatory and workflow integration, and demonstration of meaningful clinical benefit, while ensuring that AI complements rather than replaces clinical judgment, professional accountability, and patient-centered care.

## 6 Recommendations

Future AI development in surgery should place greater emphasis on clinical utility and decision support rather than model development alone. AI systems should be designed around clearly defined perioperative decision points, including surgical candidacy, treatment selection, intraoperative intervention thresholds, and postoperative disposition planning. Framing AI around specific clinical decisions rather than abstract risk estimates may improve clinical relevance, usability, and adoption.

Validation requirements should be matched to the intended use of the AI tool. Models intended for local deployment may require local temporal validation, calibration monitoring, and prospective impact evaluation. In contrast, tools intended for use across multiple institutions require broader external validation across diverse populations, workflows, and healthcare settings. Regardless of the deployment context, prospective evaluation remains essential to assess clinical utility, safety, and impact on decision-making.

Future studies should increasingly evaluate decision impact, clinician behavior, workflow outcomes, and patient-centered outcomes rather than relying solely on predictive performance metrics.

The integration of AI into perioperative care should occur within a shared decision-making framework that preserves clinician oversight and patient autonomy. AI tools should facilitate transparent communication of risks, benefits, and uncertainties to support informed clinical discussions. Accordingly, interpretability and explainability should remain central design considerations across all perioperative phases.

Regulatory frameworks should continue to evolve to distinguish predictive models from clinical decision-support systems. While regulatory pathways are beginning to emerge for selected preoperative applications, clearer guidance is needed for intraoperative and postoperative AI systems. Safe implementation will require robust validation standards, human-in-the-loop oversight, ongoing performance monitoring, and post-deployment surveillance.

Implementation challenges remain substantial and include workflow integration, interoperability, user acceptance, infrastructure requirements, and regulatory compliance. Successful deployment will depend on seamless integration with clinical workflows, electronic health records, and existing perioperative care pathways, while minimizing disruption to routine clinical practice.

Ethical and equity considerations should remain central throughout AI development and implementation. AI systems should be evaluated for bias across demographic and clinical subgroups, particularly when informing decisions related to treatment access, discharge planning, and rehabilitation. Transparent reporting of training data, model performance, and limitations is essential to maintain trust and accountability.

Finally, successful translation of AI-enabled surgical decision support into routine practice will require multidisciplinary collaboration among clinicians, data scientists, engineers, ethicists, healthcare administrators, and regulators. Such collaboration is essential to ensure scientific rigor, ethical integrity, clinical relevance, and safe implementation.

## 7 Limitations

This review has some limitations. First, although focused on surgical decision-making, much of the available literature remains prediction-oriented, with limited evidence demonstrating the direct impact of AI on clinical decisions, clinician behavior, or patient outcomes.

Second, the search strategy was intentionally designed to identify AI applications explicitly linked to surgical decision-making and perioperative implementation. Consequently, technically oriented AI studies without a clear clinical decision-support context may not have been captured. Therefore, this review should be interpreted as a focused scoping review of AI-supported surgical decision-making rather than an exhaustive review of all AI applications in surgery.

Third, substantial heterogeneity existed across studies in terms of clinical context, outcomes, data sources, methodologies, and implementation settings, limiting comparability and precluding quantitative synthesis.

Fourth, validation quality was inconsistent. Many studies relied on retrospective, single-center datasets, and relatively few evaluated AI tools in prospective clinical environments or assessed their impact on real-world decision-making. Furthermore, validation requirements vary according to the intended use of the AI system, making direct comparison across studies challenging.

Fifth, regulatory and implementation details were frequently underreported, making it difficult to distinguish experimental models from clinically deployable systems.

Sixth, data quality and bias remain important concerns, as many studies relied on imperfect datasets and reported limited subgroup analyses, raising issues related to fairness, equity, and generalizability.

Seventh, the inclusion of both primary and secondary evidence sources may introduce overlap because review articles can summarize primary studies represented elsewhere in the evidence base. We therefore classified all sources according to evidence type, interpreted primary and secondary evidence separately, and avoided quantitative pooling across the two groups. Accordingly, counts of included publications should not be interpreted as counts of independent datasets or independent confirmations of an effect.

Finally, publication bias may have influenced the available evidence, as studies reporting favorable AI performance are more likely to be published than studies reporting negative or inconclusive findings.

Despite these limitations, this review provides a clinically grounded synthesis of current AI applications in surgical decision-making and identifies important methodological, implementation, and translational gaps that must be addressed to support safe, effective, and equitable adoption in surgical practice.
