# Supplementary material for: Artificial intelligence in surgical decision-making across the perioperative continuum: a scoping review
Source: Front Digit Health. 2026 Sep 3;8:1871727. doi: 10.3389/fdgth.2026.1871727 (PMC13581544; doi:10.3389/fdgth.2026.1871727)
Supplement: Supplementary file 1 [file Datasheet1.pdf]

Supplementary Table S2. Complete Search Strategies Used for Study Identification

Date searched: 31 January 2026

Search period: 1 January 2015 – 31 January 2026

Objective: To identify studies evaluating artificial intelligence (AI) applications that support surgical decision-making across the perioperative continuum.

Database: PubMed/MEDLINE

Records retrieved: 65

Search Query

("artificial intelligence"[Title/Abstract] OR

"machine learning"[Title/Abstract] OR

"deep learning"[Title/Abstract] OR

radiomics[Title/Abstract] OR

"computer vision"[Title/Abstract] OR

"neural network*"[Title/Abstract] OR

"natural language processing"[Title/Abstract] OR

"large language model*"[Title/Abstract])

AND

(surgery[Title/Abstract] OR

surgical[Title/Abstract] OR

"surgical decision-making"[Title/Abstract] OR

"surgical planning"[Title/Abstract] OR

perioperative[Title/Abstract] OR

intraoperative[Title/Abstract] OR

postoperative[Title/Abstract] OR

"clinical decision support"[Title/Abstract] OR

"decision support system*"[Title/Abstract] OR

"decision-making"[Title/Abstract])

Filters Applied: * English language ,* Human studies,* Publication period: January 2015 to January 2026

Platform: PubMed/MEDLINE (National Library of Medicine)
Field restriction: Title/Abstract fields only
Filters: English language; Human studies; Publication period January 2015–January 2026

Note: Broader exploratory searches retrieved substantially larger numbers of records. The reported yield (n = 65) reflects records retained after application of predefined eligibility-focused screening and relevance assessment procedures specific to the review objective.

Database: Scopus

Records retrieved: 10

Search Query

("artificial intelligence" OR

"machine learning" OR

"deep learning" OR

radiomics OR

"computer vision" OR

"neural network*" OR

"natural language processing" OR

"large language model*")

AND

(surgery OR

surgical OR

"surgical decision-making" OR

"surgical planning" OR

perioperative OR

intraoperative OR

postoperative OR

"clinical decision support" OR

"decision support system*" OR

"decision-making")

AND PUBYEAR > 2014

AND PUBYEAR < 2027

AND LANGUAGE (english)

Platform: Elsevier Scopus
Field restriction: Title, Abstract, and Keywords
Filters: English language; Publication period January 2015–January 2026

Note: Initial retrieval identified a substantially larger pool of potentially relevant records. The reported yield (n = 10) reflects records retained following application of the predefined review scope, database filters, and relevance assessment procedures.

Database: Google Scholar

Records retrieved: 5

Search Query

("artificial intelligence" OR

"machine learning" OR

"deep learning" OR

radiomics OR

"computer vision" OR

"neural network" OR

"natural language processing" OR

"large language model")

AND

(surgery OR

surgical OR

"surgical decision-making" OR

"surgical planning" OR

perioperative OR

intraoperative OR

postoperative OR

"clinical decision support" OR

"decision support system" OR

"decision-making")

Google Scholar Screening Procedure

Google Scholar results were sorted by relevance. The first **100 records (10 result pages)** were screened sequentially according to relevance ranking. Duplicate records and clearly irrelevant publications were excluded before full-text assessment. Screening was discontinued after **three consecutive result pages yielded no potentially eligible studies**. Full texts of potentially eligible studies were reviewed against the predefined inclusion and exclusion criteria. A total of **5 eligible studies** were identified through the Google Scholar search process. Reference lists of eligible studies and relevant review articles were also manually screened to identify additional relevant publications not captured through database searching.

Additional Search Procedures

* Reference lists of eligible studies were manually screened for additional relevant publications.

* Reference lists of relevant systematic reviews, narrative reviews, and scoping reviews were searched to identify potentially eligible studies not captured through database searching.

* Duplicate records were removed before screening.

* The search strategy was intentionally designed to identify studies explicitly addressing AI-supported surgical decision-making across the perioperative continuum rather than all AI applications in surgery.
